# Ethyl 4-(dimethyl­amino)benzoate

**DOI:** 10.1107/S160053680905541X

**Published:** 2010-01-13

**Authors:** J. Kalyana Sundar, V. Natarajan, M. Arivanandhan, Y. Hayakawa, S. Natarajan

**Affiliations:** a Department of Physics, Madurai Kamaraj University, Madurai 625 021, India; b Department of Physics, Aditanar College of Arts and Science, Tiruchendur 628 216, India; c Nanodevices and Nanomaterials Division, Research Institute of Electronics, Shizuoka University, Hamamatsu 432 8011, Japan

## Abstract

Mol­ecules of the title compound, C_11_H_15_NO_2_, are essentially planar (r.m.s. deviation = 0.035 Å) and are linked into a chain along the *a* axis by weak C—H⋯O hydrogen bonds.

## Related literature

Benzoic acid and its derivatives are good inhibitors of influenza viruses, see: Luo *et al.* (1995 ▶). For the use of benzoic acid derivatives such as 4-amino­benzoic acid as bifunctional organic ligands due to the variety of their coordination modes, see: Amiraslanov *et al.* (1979 ▶); Chen & Chen (2002 ▶); Hauptmann *et al.* (2000 ▶). For the use of the title compound as a part of a self-curing two-part system comprising degradable copolymers with applications in medicine and dentistry as root-canal sealants, root-canal filling materials, dental restorative materials, implant materials, bone cements and pulp-capping materials, see: Jia & Jin (2004 ▶).
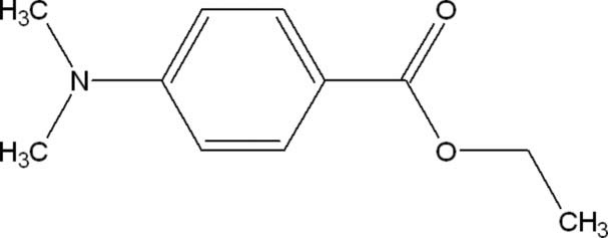


## Experimental

### 

#### Crystal data

C_11_H_15_NO_2_
*M* *_r_* = 193.24
Monoclinic, 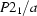

*a* = 12.6949 (8) Å
*b* = 6.6596 (4) Å
*c* = 12.8529 (9) Å
β = 98.672 (11)°
*V* = 1074.20 (12) Å^3^
*Z* = 4
Mo *K*α radiation
μ = 0.08 mm^−1^
*T* = 293 K
0.18 × 0.15 × 0.13 mm

#### Data collection

Nonius MACH-3 diffractometer
Absorption correction: ψ scan (North *et al.*, 1968 ▶) *T*_min_ = 0.985, *T*_max_ = 0.989
4088 measured reflections
1873 independent reflections
1424 reflections with *I* > 2σ(*I*)
*R*_int_ = 0.051
3 standard reflections every 60 min  intensity decay: none

#### Refinement

*R*[*F* ^2^ > 2σ(*F* ^2^)] = 0.043
*wR*(*F* ^2^) = 0.134
*S* = 1.05
1873 reflections
131 parameters
H-atom parameters constrained
Δρ_max_ = 0.16 e Å^−3^
Δρ_min_ = −0.14 e Å^−3^

### 

Data collection: *CAD-4 EXPRESS* (Enraf–Nonius, 1994 ▶); cell refinement: *CAD-4 EXPRESS*; data reduction: *XCAD4* (Harms & Wocadlo, 1996 ▶); program(s) used to solve structure: *SHELXS97* (Sheldrick, 2008 ▶); program(s) used to refine structure: *SHELXL97* (Sheldrick, 2008 ▶); molecular graphics: *PLATON* (Spek, 2009 ▶); software used to prepare material for publication: *SHELXL97*.

## Supplementary Material

Crystal structure: contains datablocks global, I. DOI: 10.1107/S160053680905541X/ci2998sup1.cif

Structure factors: contains datablocks I. DOI: 10.1107/S160053680905541X/ci2998Isup2.hkl

Additional supplementary materials:  crystallographic information; 3D view; checkCIF report

## Figures and Tables

**Table 1:** Hydrogen-bond geometry (Å, °)

*D*—H⋯*A*	*D*—H	H⋯*A*	*D*⋯*A*	*D*—H⋯*A*
C2—H2⋯O2^i^	0.93	2.55	3.4682 (19)	168

Symmetry code: (i) 
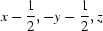
.

**Fig. 1.**
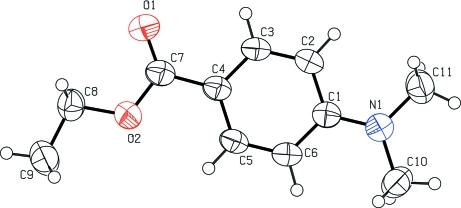
The molecular structure of the title compound, showing 30% probability displacement ellipsoids and the atom-numbering scheme.

## supplementary crystallographic information

### Comment

Benzoic acid and its derivatives are good inhibitors of influenza viruses (Luo *et al.*, 1995). Some benzoic acid derivatives such as 4-aminobenzoic acid have been extensively reported in coordination chemistry as bifunctional organic ligands due to the varieties of their coordination modes (Chen & Chen, 2002; Amiraslanov *et al.*, 1979; Hauptmann *et al.*, 2000). The title compound, a tertiary amine, is used as a part of self-curing two part system for dental/ medical compositions comprising degradable copolymers which are suitable for use as root canal sealants, root canal filling materials, dental restorative materials, implant materials, bone cements and pulp capping materials (Jia *et al.*, 2004).

The molecule of the title compound, C_11_H_15_N O_2_, is essentially planar (r.m.s. deviation 0.035 Å). The molecules are linked into a chain along the *a* axis by weak C—H···O hydrogen bonds.

### Experimental

Ethyl 4-(dimethylamino)benzoate (EDMAB) obtained from Sigma–Aldrich, India, was dissolved in ethanol. The saturated solution was transferred to a crystallizer and covered by a perforated polyethylene sheet for controlled evaporation at room temperature. Colourless crystals were harvested, after five days

### Refinement

H atoms were placed at calculated positions and allowed to ride on their carrier atoms, with C-H = 0.93–0.97 Å and *U*_iso_ = 1.2*U*_eq_(C) for CH_2_ and CH groups and *U*_iso_ = 1.5*U*_eq_(C) for CH_3_ group.

### Crystal data

**Table:**

C_11_H_15_NO_2_	*F*(000) = 416
*M**_r_* = 193.24	*D*_x_ = 1.195 Mg m^−^^3^
Monoclinic, *P*2_1_/*a*	Mo *K*α radiation, λ = 0.71073 Å
Hall symbol: -P 2yab	Cell parameters from 25 reflections
*a* = 12.6949 (8) Å	θ = 2–25°
*b* = 6.6596 (4) Å	µ = 0.08 mm^−^^1^
*c* = 12.8529 (9) Å	*T* = 293 K
β = 98.672 (11)°	Block, colourless
*V* = 1074.20 (12) Å^3^	0.18 × 0.15 × 0.13 mm
*Z* = 4	

### Data collection

**Table:**

Nonius MACH-3 diffractometer	1424 reflections with *I* > 2σ(*I*)
Radiation source: fine-focus sealed tube	*R*_int_ = 0.051
graphite	θ_max_ = 25.0°, θ_min_ = 3.2°
ω–2θ scans	*h* = −1→15
Absorption correction: ψ scan (North et al., 1968)	*k* = −7→7
*T*_min_ = 0.985, *T*_max_ = 0.989	*l* = −15→15
4088 measured reflections	3 standard reflections every 60 min
1873 independent reflections	intensity decay: none

### Refinement

**Table:**

Refinement on *F*^2^	Secondary atom site location: difference Fourier map
Least-squares matrix: full	Hydrogen site location: inferred from neighbouring sites
*R*[*F*^2^ > 2σ(*F*^2^)] = 0.043	H-atom parameters constrained
*wR*(*F*^2^) = 0.134	*w* = 1/[σ^2^(*F*_o_^2^) + (0.0618*P*)^2^ + 0.2249*P*] where *P* = (*F*_o_^2^ + 2*F*_c_^2^)/3
*S* = 1.05	(Δ/σ)_max_ = 0.001
1873 reflections	Δρ_max_ = 0.16 e Å^−^^3^
131 parameters	Δρ_min_ = −0.13 e Å^−^^3^
0 restraints	Extinction correction: *SHELXL97* (Sheldrick, 2008), Fc^*^=kFc[1+0.001xFc^2^λ^3^/sin(2θ)]^-1/4^
Primary atom site location: structure-invariant direct methods	Extinction coefficient: 0.018 (3)

### Special details

**Table:**

Geometry. All esds (except the esd in the dihedral angle between two l.s. planes) are estimated using the full covariance matrix. The cell esds are taken into account individually in the estimation of esds in distances, angles and torsion angles; correlations between esds in cell parameters are only used when they are defined by crystal symmetry. An approximate (isotropic) treatment of cell esds is used for estimating esds involving l.s. planes.
Refinement. Refinement of *F*^2^ against ALL reflections. The weighted *R*-factor *wR* and goodness of fit *S* are based on *F*^2^, conventional *R*-factors *R* are based on *F*, with *F* set to zero for negative *F*^2^. The threshold expression of *F*^2^ > σ(*F*^2^) is used only for calculating *R*-factors(gt) *etc*. and is not relevant to the choice of reflections for refinement. *R*-factors based on *F*^2^ are statistically about twice as large as those based on *F*, and *R*- factors based on ALL data will be even larger.

### Fractional atomic coordinates and isotropic or equivalent isotropic displacement parameters (Å^2^)

**Table:**

	*x*	*y*	*z*	*U*_iso_*/*U*_eq_	
O1	0.40072 (9)	0.08183 (18)	0.88044 (9)	0.0572 (4)	
O2	0.54776 (10)	0.0855 (2)	0.80386 (11)	0.0707 (4)	
C1	0.34843 (11)	−0.5441 (3)	0.66547 (12)	0.0469 (4)	
C4	0.42325 (12)	−0.1813 (3)	0.76552 (12)	0.0461 (4)	
N1	0.31326 (11)	−0.7203 (3)	0.61807 (13)	0.0627 (5)	
C7	0.46496 (12)	0.0065 (3)	0.81690 (12)	0.0497 (4)	
C3	0.32462 (12)	−0.2619 (3)	0.77847 (12)	0.0479 (4)	
H3	0.2828	−0.1950	0.8208	0.057*	
C6	0.44776 (12)	−0.4608 (3)	0.65238 (13)	0.0528 (5)	
H6	0.4900	−0.5262	0.6098	0.063*	
C2	0.28788 (12)	−0.4375 (3)	0.73023 (13)	0.0491 (4)	
H2	0.2217	−0.4869	0.7405	0.059*	
C5	0.48322 (12)	−0.2852 (3)	0.70133 (13)	0.0531 (5)	
H5	0.5492	−0.2343	0.6913	0.064*	
C8	0.43523 (14)	0.2641 (3)	0.93720 (13)	0.0574 (5)	
H8A	0.4448	0.3713	0.8884	0.069*	
H8B	0.5023	0.2419	0.9829	0.069*	
C1A	0.37347 (16)	−0.8242 (3)	0.54815 (15)	0.0685 (5)	
H1A1	0.3762	−0.7437	0.4867	0.103*	
H1A2	0.3397	−0.9501	0.5277	0.103*	
H1A3	0.4445	−0.8481	0.5835	0.103*	
C2A	0.21248 (14)	−0.8068 (3)	0.63395 (17)	0.0690 (6)	
H2A1	0.2090	−0.8138	0.7080	0.103*	
H2A2	0.2062	−0.9395	0.6044	0.103*	
H2A3	0.1553	−0.7247	0.6000	0.103*	
C9	0.34993 (18)	0.3176 (3)	1.00051 (16)	0.0767 (6)	
H9A	0.2835	0.3340	0.9545	0.115*	
H9B	0.3683	0.4409	1.0376	0.115*	
H9C	0.3431	0.2125	1.0502	0.115*	

### Atomic displacement parameters (Å^2^)

**Table:**

	*U*^11^	*U*^22^	*U*^33^	*U*^12^	*U*^13^	*U*^23^
O1	0.0615 (7)	0.0553 (8)	0.0586 (7)	−0.0047 (6)	0.0215 (6)	−0.0083 (6)
O2	0.0583 (7)	0.0663 (9)	0.0929 (10)	−0.0141 (7)	0.0289 (7)	−0.0111 (7)
C1	0.0436 (8)	0.0519 (10)	0.0451 (8)	0.0024 (7)	0.0062 (6)	0.0041 (7)
C4	0.0448 (8)	0.0495 (10)	0.0459 (8)	0.0027 (7)	0.0129 (7)	0.0052 (7)
N1	0.0543 (8)	0.0650 (11)	0.0710 (10)	−0.0081 (7)	0.0163 (7)	−0.0160 (8)
C7	0.0481 (8)	0.0529 (10)	0.0500 (9)	0.0034 (8)	0.0132 (7)	0.0061 (8)
C3	0.0465 (8)	0.0507 (10)	0.0500 (9)	0.0056 (7)	0.0186 (7)	0.0041 (7)
C6	0.0470 (8)	0.0591 (11)	0.0560 (10)	0.0024 (8)	0.0200 (7)	−0.0052 (8)
C2	0.0399 (8)	0.0560 (11)	0.0535 (9)	0.0010 (7)	0.0139 (7)	0.0055 (8)
C5	0.0431 (8)	0.0622 (12)	0.0578 (10)	−0.0045 (8)	0.0198 (7)	−0.0025 (8)
C8	0.0711 (11)	0.0483 (10)	0.0526 (10)	−0.0032 (9)	0.0089 (8)	−0.0005 (8)
C1A	0.0794 (13)	0.0657 (13)	0.0612 (11)	0.0003 (11)	0.0133 (9)	−0.0123 (10)
C2A	0.0613 (11)	0.0597 (12)	0.0851 (13)	−0.0125 (10)	0.0083 (9)	−0.0028 (10)
C9	0.0951 (15)	0.0653 (13)	0.0750 (13)	0.0003 (12)	0.0296 (11)	−0.0139 (11)

### Geometric parameters (Å, °)

**Table:**

O1—C7	1.3361 (19)	C2—H2	0.9300
O1—C8	1.449 (2)	C5—H5	0.9300
O2—C7	1.2095 (19)	C8—C9	1.493 (2)
C1—N1	1.365 (2)	C8—H8A	0.9700
C1—C2	1.408 (2)	C8—H8B	0.9700
C1—C6	1.411 (2)	C1A—H1A1	0.9600
C4—C5	1.389 (2)	C1A—H1A2	0.9600
C4—C3	1.395 (2)	C1A—H1A3	0.9600
C4—C7	1.475 (3)	C2A—H2A1	0.9600
N1—C1A	1.442 (2)	C2A—H2A2	0.9600
N1—C2A	1.446 (2)	C2A—H2A3	0.9600
C3—C2	1.372 (2)	C9—H9A	0.9600
C3—H3	0.9300	C9—H9B	0.9600
C6—C5	1.372 (3)	C9—H9C	0.9600
C6—H6	0.9300		
			
C7—O1—C8	117.15 (13)	O1—C8—C9	106.59 (14)
N1—C1—C2	121.76 (14)	O1—C8—H8A	110.4
N1—C1—C6	121.51 (14)	C9—C8—H8A	110.4
C2—C1—C6	116.73 (16)	O1—C8—H8B	110.4
C5—C4—C3	117.49 (16)	C9—C8—H8B	110.4
C5—C4—C7	119.76 (14)	H8A—C8—H8B	108.6
C3—C4—C7	122.75 (14)	N1—C1A—H1A1	109.5
C1—N1—C1A	121.48 (15)	N1—C1A—H1A2	109.5
C1—N1—C2A	121.10 (15)	H1A1—C1A—H1A2	109.5
C1A—N1—C2A	117.40 (16)	N1—C1A—H1A3	109.5
O2—C7—O1	123.00 (17)	H1A1—C1A—H1A3	109.5
O2—C7—C4	124.59 (15)	H1A2—C1A—H1A3	109.5
O1—C7—C4	112.41 (13)	N1—C2A—H2A1	109.5
C2—C3—C4	121.61 (15)	N1—C2A—H2A2	109.5
C2—C3—H3	119.2	H2A1—C2A—H2A2	109.5
C4—C3—H3	119.2	N1—C2A—H2A3	109.5
C5—C6—C1	121.20 (14)	H2A1—C2A—H2A3	109.5
C5—C6—H6	119.4	H2A2—C2A—H2A3	109.5
C1—C6—H6	119.4	C8—C9—H9A	109.5
C3—C2—C1	121.24 (15)	C8—C9—H9B	109.5
C3—C2—H2	119.4	H9A—C9—H9B	109.5
C1—C2—H2	119.4	C8—C9—H9C	109.5
C6—C5—C4	121.73 (15)	H9A—C9—H9C	109.5
C6—C5—H5	119.1	H9B—C9—H9C	109.5
C4—C5—H5	119.1		
			
C2—C1—N1—C1A	177.23 (16)	C7—C4—C3—C2	−179.64 (15)
C6—C1—N1—C1A	−3.2 (3)	N1—C1—C6—C5	−179.15 (16)
C2—C1—N1—C2A	−1.0 (3)	C2—C1—C6—C5	0.5 (2)
C6—C1—N1—C2A	178.53 (16)	C4—C3—C2—C1	0.1 (2)
C8—O1—C7—O2	−1.5 (2)	N1—C1—C2—C3	179.19 (15)
C8—O1—C7—C4	178.51 (13)	C6—C1—C2—C3	−0.4 (2)
C5—C4—C7—O2	3.6 (3)	C1—C6—C5—C4	−0.2 (3)
C3—C4—C7—O2	−176.55 (16)	C3—C4—C5—C6	−0.2 (2)
C5—C4—C7—O1	−176.49 (14)	C7—C4—C5—C6	179.69 (15)
C3—C4—C7—O1	3.4 (2)	C7—O1—C8—C9	179.26 (15)
C5—C4—C3—C2	0.3 (2)		

### Hydrogen-bond geometry (Å, °)

**Table:**

*D*—H···*A*	*D*—H	H···*A*	*D*···*A*	*D*—H···*A*
C2—H2···O2^i^	0.93	2.55	3.4682 (19)	168

Symmetry codes: (i) *x*−1/2, −*y*−1/2, *z*.
